# Impact of fasting on lipid profile assessment of Brazilian adults

**DOI:** 10.20945/2359-4292-2025-0195

**Published:** 2025-09-28

**Authors:** Vitor Emanuel Nunes Pinto, Paulo Caleb Júnior Lima Santos, Enildo Broetto Pimentel, José Geraldo Mill, Rafael de Oliveira Alvim

**Affiliations:** 1 Faculdade de Medicina, Universidade Federal do Amazonas, Manaus, AM, Brasil; 2 Departamento de Farmacologia, Escola Paulista de Medicina, Universidade Federal de São Paulo, São Paulo, SP, Brasil; 3 Departamento de Ciências Fisiológicas, Universidade Federal do Espírito Santo, Vitória, ES, Brasil; 4 Departamento de Ciências Fisiológicas, Universidade Federal do Amazonas, Manaus, AM, Brasil

**Keywords:** Lipid profile, dyslipidemia, fasting, adults

## Abstract

**Objective:**

Dyslipidemia is a common cardiovascular risk factor, with ongoing debate over whether lipid profile assessment, with or without fasting, affects the accuracy of cardiovascular risk evaluation. The objective of this study is to evaluate the effect of fasting status on lipid profile values and the prevalence of dyslipidemia.

**Subjects and methods:**

A total of 269 adults (20-69 years) from Vitória-ES (Brazil) were included. Two blood samples were collected on the same day: one in the morning after a 10-12-hour fast and the other in the afternoon, post-lunch (1-5 pm). Dyslipidemias were classified according to the Brazilian Guidelines.

**Results:**

The percentage of participants classified with low HDL-c (male: 54.2 vs. 38.2%, p < 0.001; female: 29.7 vs. 15.2%, p < 0.001) and hypertriglyceridemia (male: 59.5 vs. 26.7%, p < 0.001; female: 50.0 vs. 22.5%, p < 0.001) was higher in the non-fasting state. Furthermore, HDL-c levels were higher in after fasting. Triglyceride levels were higher in the non-fasting state, while LDL-c concentrations were slightly reduced in the non-fasting state. Without fasting, 85 individuals previously classified as having normal TG were reclassified as having hypertriglyceridemia, and 41 individuals previously classified as having normal HDL-c were reclassified as having low HDL-c.

**Conclusion:**

The feeding state is key to detecting and managing dyslipidemias, especially hypertriglyceridemia and low HDL-c. Removing the fasting requirement could improve cardiovascular risk identification, increase patient adherence to testing and treatment. However, the significant differences in the lipid profile concentrations must be considered in the patient’s management in the clinical practice.

## INTRODUCTION

Cardiovascular diseases (CVD) represent the main cause of mortality and reduced quality of life in Brazil and worldwide (^1^). In 2022, an estimated 19.8 million people died from CVD globally (^2^). In Brazil, in 2019, more than 400,000 deaths were attributed to these diseases, with an age-standardized prevalence and incidence of 5,454 and 475 cases per 100,000 inhabitants, respectively (^2^). In recent decades, several epidemiological studies have shown that the most prevalent risk factors for CVD are: aging (^3^), high blood pressure (^4^), diabetes mellitus (^5^), obesity (^6^), sedentary lifestyle (^7^), genetics (^8^), smoking (^9^) and dyslipidemia (^10^).

Dyslipidemia is a metabolic condition characterized by abnormal levels of lipids in the blood (^10^). Increased serum levels of low-density lipoprotein (LDL-c) substantially increase the risk of developing coronary artery disease and stroke (^11^,^12^). Furthermore, a low concentration of high-density lipoprotein (HDL-c) and high levels of triglycerides (TG) also contribute significantly to the development of CVD (^13^,^14^). Consequently, the control of the lipid profile is essential for the prevention and treatment of CVD (^15^).

Reference values for all these lipids were established for fasting blood collection, as the occurrence of serum lipid profile depends on the time elapsed and the amount of food ingested, which can increase TG concentrations and impact the estimation of LDL-c in particular (^16^). However, the scientific community has discussed the need for fasting, given that individuals spend most of their lives in a postprandial state (^17^,^18^). Therefore, determining the lipid profile without fasting could have better predictive power for CVD (^19^). As a result, health organizations in several countries are updating their guidelines and adopting the random lipid profile without fasting in their recommendations (^20^). In Brazil, some scientific societies have recommended making fasting more flexible, as it also benefits patients who have some type of dietary limitation, such as individuals with diabetes, the elderly, children and pregnant women, in addition to promoting greater practicality in the work routine of clinical analysis laboratories (^15^).

In this context, there is an ongoing debate about whether fasting is essential for testing lipid profile and how this factor would impact the reference values for normality and/or individuals’ goals. Therefore, the main aim of this study was to evaluate the impact of fasting status on the lipid profile of adults and the prevalence of dyslipidemia.

## SUBJECTS AND METHODS

### Study population

In 2013, 318 volunteers (residents of Vitória, ES, Brazil) were recruited from a random sample of households drawn from 20 census tracts of the city. The houses were visited to include one volunteer (20-69 years old) per residence. Recruitment was done on a quota basis, with a target of 50% for each sex and 20% per decade of age. The following individuals were not included: those with acute illnesses; those who were bedridden or had reduced mobility; those with communication difficulties; and women who were pregnant or were breastfeeding. In this study, 269 volunteers were eligible because 49 were using lipid-lowering drugs and were therefore excluded. The project was approved by the Research Ethics Committee of the Health Sciences Center of the Federal University of Espírito Santo (UFES, Protocol 201.110) and all participants signed a consent form. Research assistants collected sociodemographic data at home (gender, self-reported race/color, monthly income and schooling), as well as information on physical activity, regularly-used medications and lifestyle habits (smoking). Following this, they scheduled the day of the blood test to determine lipid fractions at the Cardiovascular Research Clinic of the Federal University of Espírito Santo Hospital. Participants were instructed not to stop taking medication and to maintain their eating habits. The day before the blood was taken, they fasted after 8 p.m. and abstained from alcohol consumption and vigorous physical exercise. Blood samples were collected the following day at 8 a.m., following an overnight fast of a minimum of 12 hours.

### Anthropometric variables

Body mass was measured using a digital scale (Toledo Scale, Brazil – 0.05 kg accuracy) with the individuals barefoot wearing only underwear. Height was determined using a wall-mounted stadiometer (Seca Stadiometer; Co, Hamburg, Germany – 0.1 cm accuracy). Waist circumference was measured at the mean point between the lowest rib margin and the iliac crest. Body mass index (BMI) was calculated as the ratio between weight in kilograms and the square of their height in meters (kg/m^2^).

### Blood pressure assessment

Systolic blood pressure (SBP) and diastolic blood pressure (DBP) were measured by the oscillometric method using an OMRON digital manometer, model HEM-741CINT. The cuff was placed on the left arm after resting for five minutes. Three blood pressure measurements were taken, with a 3-minute interval between each measurement. Patients with SBP ≥ 140 and/or DBP ≥ 90 mmHg, or those taking antihypertensive medication, were considered hypertensive (^21^).

### Classification of Diabetes and Metabolic Syndrome (MetS)

The diagnosis of diabetes was established in the presence of fasting glucose ≥ 126 mg/dL, glycated hemoglybin ≥ 6.5%, and/or the use of antidiabetic drugs (^22^). For MetS, the Joint Interim Statement criteria were used (^23^). This requires the presence of any three of the following five risk factors: high blood pressure (systolic blood pressure ≥ 130 mmHg and/or diastolic blood pressure ≥ 85 mmHg, or drug treatment [antihypertensive]); high fasting glucose (≥100 mg/dL or drug treatment [antidiabetic]); low HDL-c (<40 mg/dL for men and <50 mg/dL for women, or drug treatment [lipid-lowering]); high TG (≥150 mg/dL or drug treatment [lipid-lowering]); and high WC (≥90 cm in men and ≥80 cm in women), considering the International Diabetes Federation cut-off point for the South American population.

### Lipid profile assessment

Two blood samples were taken on the same day, one in the early morning (around 8 a.m.) with the participant fasting for approximately 12 hours, and the other in the afternoon after lunch (between 1 and 5 pm). Blood was collected by venipuncture in the forearm and the samples were processed at the collection site and sent to a central laboratory (Tommasi Laboratório, Vitória, ES, Brazil) where serum concentrations of total cholesterol, HDL-c and TG were determined. TG was measured using the glycerol-phosphate oxidase colorimetric method; total cholesterol was measured by a standard enzymatic colorimetric method; and HDL-c was measured using a direct homogeneous enzymatic method. LDL-c was calculated using the Friedewald equation for participants with TG concentrations ≤ 400 mg/dL. Non-HDL cholesterol was calculated by subtracting HDL-c from total cholesterol. Hypercholesterolemia was defined as total cholesterol ≥ 190 mg/dL. Hypertriglyceridemia was defined as TG ≥ 150 mg/dL in fasting and ≥ 175 mg/dL in non-fasting. High LDL-c was defined as LDL-c ≥ 130 mg/dL. Low HDL-c was defined as HDL-c ≤ 40 mg/dL. High non-HDL-c cholesterol was defined as non-HDL-c cholesterol ≥ 160 mg/dL (^10^).

### Statistical analysis

The data are described as mean and standard deviation for the continuous variables and as count and percentage for the categorical variables. The normality of the continuous variables was tested with the Kolmogorov-Smirnov test. For variables with a normal distribution, the difference in means between the two conditions (fasting and non-fasting) was determined using paired measures t-test and the data were presented as mean and standard deviation. However, for variables that did not have a normal distribution, the Wilcoxon test was used to determine the difference in medians between the two conditions (fasting and non-fasting) and the data was presented as median and interquartile range. The relative and absolute changes between the groups with normal and altered TG and HDL levels were compared using the Mann-Whitney U test. McNemar’s test was used to compare the differences in the proportion of dyslipidemia during fasting and non-fasting. All statistical procedures were conducted with SPSS version 24.0 statistical package (SPSS Inc., Chicago, Illinois, USA).

## RESULTS

**Table 1** shows the general characteristics of the sample stratified by sex. The average age of the participants was 43.7 ± 13.9 years, and 34.4% of men and 28.3% of women have MetS.

**Table 1:** General characteristics of the sample stratified by sex

	Male	Female
N	131	138
Age (years)	43.4 ± 14.0	44.0 ± 13.8
BMI (kg/m^2^)	26.7 ± 4.6	26.9 ± 5.3
WC (cm)	93.4 ± 14.5	86.7 ± 12.3
SBP (mmHg)	125.3 ± 14.7	119.1 ± 18.2
DBP (mmHg)	76.9 ± 9.8	75.3 ± 9.3
Fasting glucose (mg/dL)	94.5 ± 18.3	95.2 ± 30.0
Race/color, n (%)		
White	54 (41.2)	61 (44.2)
Brown	59 (45.0)	61 (44.2)
Black	17 (13.0)	14 (10.2)
Others	1 (0.8)	2 (1.4)
Obesity, n (%)	24 (18.3)	37 (26.8)
Hypertension, n (%)	36 (27.5)	34 (24.6)
Diabetes, n (%)	6 (4.6)	12 (8.7)
Metabolic Syndrome, n (%)	45 (34.4)	39 (28.3)

Continuous data are expressed as mean ± standard deviation and categorical data as absolute number (percentage). BMI: body mass index; WC: waist circumference; SBP: systolic blood pressure; DBP: diastolic blood pressure.

**Table 2** shows the comparison of lipid variables in the fasting and non-fasting states. In both female and male subjects, LDL-c and HDL-c levels were higher in fasting when compared to non-fasting state. In contrast, VLDL-c, Non-HDL-c cholesterol and TG showed higher levels during non-fasting state when compared to fasting state. There were no differences in TC between the two states for both sexes.

**Table 2:** Comparison of lipid variables in the fasting and nonfasting states

Variables	Fasting	Nonfasting	p-value
**Male**			
TC, mg/dL*	189.9 ± 40.6	189.1 ± 43.9	0.559
LDL-c, mg/dL*	119.5 ± 37.3	115.3 ± 38.0	<0.001
HDL-c, mg/dL#	43.0 (36.0-50.0)	38.0 (33.0-45.0)	<0.001
VLDL-c, mg/dL#	23.0 (21.0-31.0)	37.0 (29.0-40.0)	<0.001
N-HDL-c, mg/dL*	145.7 ± 40.0	148.7 ± 43.0	0.015
TG, mg/dL#	110.0 (84.0-157.0)	207.0 (144.0-280.0)	<0.001
**Female**			
TC, mg/dL*	186.1 ± 39.9	187.0 ± 41.1	0.373
LDL-c, mg/dL#	110.0 (80.8-134.0)	107.5 (80.0-131.0)	0.012
HDL-c, mg/dL#	50.0 (42.8-57.0)	46.0 (38.0-53.0)	<0.001
VLDL-c, mg/dL#	23.5 (20.0-29.0)	34.0 (24.0-38.3)	<0.001
N-HDL-c, mg/dL*	135.3 ± 38.2	139.9 ± 39.1	<0.001
TG, mg/dL#	111.5 (79.0-143.3)	175.0 (114.0-247.0)	<0.001

TC: total cholesterol; LDL-c: low-density lipoprotein cholesterol; HDL-c: high-density lipoprotein cholesterol; N-HDL: non-HDL cholesterol; VLDL: very low-density lipoprotein cholesterol; TG: triglycerides. *Student’s t-test for repeated measurements and data was presented as the mean ± standard deviation. #Wilcoxon test and data were presented as medians and interquartile ranges.

**Table 3** shows the comparison of the differences in the proportion of dyslipidemia during fasting and non-fasting. In both sexes, the percentage of participants classified as having low HDL-c and hypertriglyceridemia is higher in the non-fasting state, while there was no difference in the percentage of participants classified as having high TC and high LDL-c. The percentage of participants classified as having high non-HDL-c cholesterol was higher during non-fasting only in women.

**Table 3:** Comparison of the differences in the proportion of dyslipidemia during fasting and nonfasting

Variables	Fasting	Nonfasting	p-value
**Male**			
High TC (%)	47.3	45.0	0.508
High LDL-c (%)	37.4	32.8	0.146
High N-HDL-c (%)	32.1	36.6	0.109
Low HDL-c (%)	38.2	54.2	<0.001
Hypertriglyceridemia (%)	26.7	59.5	<0.001
**Female**			
High TC (%)	44.2	44.2	1.000
High LDL-c (%)	29.0	25.4	0.180
High N-HDL-c (%)	25.4	31.2	0.039
Low HDL-c (%)	15.2	29.7	<0.001
Hypertriglyceridemia (%)	22.5	50.0	<0.001

TC: total cholesterol; LDL-c: low-density lipoprotein cholesterol; HDL-c: high-density lipoprotein cholesterol; N-HDL: non-HDL cholesterol. Proportion differences were estimated by McNemar test.

This same analysis, when stratified by the presence or absence of MetS, shows that the frequency of both hypertriglyceridemia and low HDL-c was higher in the non-fasting state across all subgroups (data not shown). For hypertriglyceridemia, this reclassification was proportionally more pronounced in individuals without MetS. In this subgroup, the frequency increased by 34.1 percentage points (from 8.1% to 42.2%), compared to a 21.4 percentage point increase in those with MetS (from 60.7% to 82.1%).

**Figure 1:**
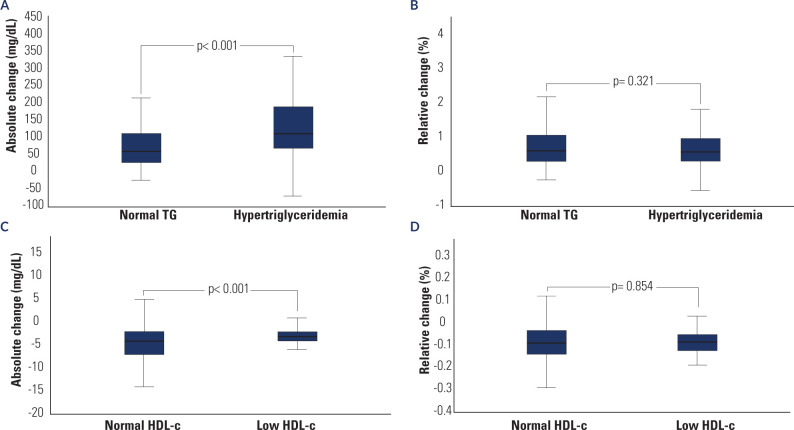
Absolute (**A** and **C**) and relative (**B** and **D**) changes in the TG and HDL-c values (mg/dL and %, respectively), according to Normal TG/Hypertriglyceridemia or to Normal HDL-c/Low-HDL-c (classified in the non-fasting state). Absolute change is calculated as non-fasting value – fasting value. Relative change is calculated as (non-fasting value – fasting value)/non-fasting value.

**Figure 1** illustrates significant differences in the absolute changes of TG and HDL-C values (A and C). In contrast, no significant differences were observed in the relative changes (B and D). In addition, without fasting, 85 individuals previously classified as having normal TG were reclassified as having hypertriglyceridemia, and 41 individuals previously classified as having normal HDL-c were reclassified as having low HDL-c.

## DISCUSSION

The main finding of this study was that the percentage of participants classified as having low HDL-c and hypertriglyceridemia was higher in the non-fasting state – with a significant number of individuals previously classified as having normal values being classified as abnormal. Additionally, HDL-c levels were higher in the fasting state, while TG showed higher levels during the non-fasting state.

Despite the acknowledged importance of this topic, the full clinical implications remain insufficiently understood (^15^,^16^). In the present study, as in the majority of clinical laboratories, LDL-c measurement was conducted using the Friedewald equation, which incorporates TG concentration in the denominator, establishing an inverse relationship (due to a subtraction). A recent systematic review (^15^) has revealed notable discrepancies among authors regarding the clinical relevance of the postprandial TG elevation. Some researchers consider this increase to be of minimal clinical significance and suggest revising reference values for the classification of hypertriglyceridemia. In contrast, others support the use of non-fasting measurements, arguing that these values more accurately reflect patients’ habitual dietary patterns. Furthermore, several studies have demonstrated that non-fasting TG levels are more strongly associated with cardiovascular events than fasting levels (^24^,^25^). In Brazil, reference values of < 150 mg/dL or < 175 mg/dL are currently used, depending on whether the patient is fasting or not, respectively (^10^). In this context, it is essential that diagnostic and pharmacotherapeutic approaches consider this metabolic dynamic, giving due attention to the influence of fasting or feeding on lipid profile assessment.

The study also found that there was an increase in non-HDL-c and a slight reduction in LDL-c concentration (due to Friedewald calculation). Non-HDL-c has been identified as a good predictor of cardiovascular disease risk, as it encompasses the cholesterol content of all atherogenic lipoproteins circulating in the bloodstream (^26^). Furthermore, its association with Apolipoprotein B (ApoB) is critical, as both play a central role in the initiation of the atherosclerotic process in arterial walls (^27^,^28^). Therefore, the evidence suggests that greater emphasis on non-HDL-c levels in lipid profile assessments may be crucial for enhancing prevention and treatment strategies in populations at risk. However, without fasting LDL-c concentrations appears to have a low variation that is clinically irrelevant, but nevertheless has implications for the diagnosis and therapeutic target in the different values of concentrations due to guidelines (such as 130, 100, 70, and 50 mg/dL). In this scenario of clinical practice, the patient’s fasting status must be observed as a potential factor of changes on lipid profile tests with repercussions on screening for diagnosis and early treatment, and on therapeutic goals (with pharmacological and non-pharmacological strategies).

In the present study, increases in the prevalence of reduced HDL-c and of hypertriglyceridemia in the postprandial state were observed. According to Endo et al. (^29^), HDL-c exerts an anti-atherosclerotic function, facilitating the reverse transport of excess cholesterol from peripheral tissues to the liver. Consequently, low HDL-c levels serve as predictors of a heightened risk for the development of cardiovascular diseases. Additionally, hypertriglyceridemia is strongly associated with an increased risk of adverse cardiovascular events, including coronary artery disease and ischemic stroke, owing to its atherogenic potential (^30^).

Therefore, the findings suggest that reliance on fasting-based assessments may fail to identify individuals at elevated cardiovascular risk. Thus, alleviating the fasting requirement for laboratory tests could provide a more comprehensive screening approach in primary health care, facilitating the early detection of dyslipidemias. Furthermore, this approach could enhance patient adherence to laboratory testing and, in turn, reduce the costs associated with screening and managing these conditions within the public healthcare system (^31^).

Physiologically, the changes observed in our study should be interpreted within the context of postprandial lipid metabolism. The acute response to a meal is characterized by postprandial lipemia, where an excursion in triglyceride-rich lipoproteins leads to peak triglyceride levels and a corresponding modest decrease in HDL-C, typically within a 3 to 6-hour window (^32^). However, the precise timing and magnitude of this peak are highly influenced by the composition of the preceding meal and individual metabolic kinetics (^33^). Consequently, although our 1:00 PM to 5:00 PM collection period aligns with this general timeframe, it introduces a methodological constraint on the interpretation of our results. A sample collected earlier in this interval may reflect a rising lipid profile, while a later sample may capture the clearance phase, creating significant heterogeneity.

Some limitations should be considered when interpreting the findings of this study. First, the sample was recruited in a single city in Brazil and the extent to which the results apply to the broader Brazilian population is unknown. Second, a standardized time interval for postprandial sample collection was not applied, although this situation also presents itself in the real world in clinical laboratories. In addition, both blood samples were collected on the same day, which should influence same patients with physiological changes. Third, our ability to control key potential confounders was limited. We did not collect data on physical activity and diet, which precluded any adjustment for these variables. Furthermore, the study’s limited sample size and consequent low statistical power restricted our ability to perform stratified analyses or assess for effect modification by other important factors, such as age, BMI and metabolic syndrome. Fourth, diabetes cases were diagnosed based on a single blood collection, which, lacking the recommended confirmatory test, may have resulted in some patient misclassification. Fifth, the lack of longitudinal follow-up precludes the evaluation of potential clinical outcomes associated with the observed variations in the lipid profile over time.

In conclusion, the feeding state plays a significant role in the detection and management of dyslipidemias, particularly regarding hypertriglyceridemia and low HDL-c. Alleviating the fasting requirement might contribute, at least partly, to improved cardiovascular risk identification throughout the day and enhance patient adherence to testing and treatment, with potentially positive implications for public health.

## Data Availability

datasets related to this article will be available upon request to the corresponding author.
